# Supplementary material for: Clinical Evaluation of a Multiplex PCR Assay for Simultaneous Detection of 18 Respiratory Pathogens in Patients with Acute Respiratory Infections
Source: Pathogens. 2022 Dec 23;12(1):21. doi: 10.3390/pathogens12010021 (PMC9862116; doi:10.3390/pathogens12010021)
Supplement: Supplementary file 1 [file pathogens-12-00021-s001.zip › pathogens-2062561-supplementary.pdf]

Pathogen	fluorescent channel	Melting temperature T_m (°C)	MPA tube NO.
IFA	FAM	52.0±1.0	Reaction tube 1
PIV-3		42.0±1.0	
IFB		33.5±1.0	
PIV-1	VIC	54.5±1.0	
CP		48.0±1.0	
MP		41.0±1.0	
PIV-2		35.0±1.0	
RSV	ROX	53.0±1.0	
PIV-4		46.0±1.0	
HRV		39.0±1.0	
HADV		31.0±1.0	
SARS-CoV-2-N	FAM	35.0±1.0	Reaction tube 2
SARS-CoV-2-ORF1ab		44.5±1.0	
SARS-CoV-2-N &SARS-CoV-21ab		35.0±1.0&44.5±1.0	
COV-HKU1	VIC	35.5±1.0	
COV-NL63		46.0±1.0	
HBOV		56.0±1.0	
HMPV	ROX	36.5±1.0	
COV-OC43		47.5±1.0	
COV-229E		56.5±1.0	

Supplementary Table S1. Fluorescent channel and Melting temperature of each respiratory pathogen probe

pathogens	Viral load (copies/mL)	Result (+/–)
H1N1（2009）、HADV-1	10^3	+
H1N1、HADV-3	10^3	+
H3N2、HADV-7	10^3	+
H5N1	10^3	+
H7N9	10^3	+
PIV1、229E	10^3	+
PIV2、HKU1	10^3	+
PIV3、OC43	10^3	+
PIV4、NL63	10^3	+
IFB-Victoria、RSV-A	10^3	+
IFB-Yamagat、RSV-B	10^3	+
HBOV、HMPV	10^3	+
MP、CP	10^3	+
HRV	10^3	+

SARS-CoV-2	10^3	+

Supplementary Table S2：Determination of the limit of detection of the MPA assay

Infection type	Pathogens	Number	Percentage (%)
Mono virus	IFA	11	11.58
	HADV	5	5.26
	HMPV	1	1.05
	229E	4	4.21
	PIV3	2	2.11
	OC43	1	1.05
	IFB	4	4.21
	RSV	4	4.21
	HRV	5	5.26
	CP	1	1.05
	MP	7	7.37
	COVID-19	26	27.37
Two viruses	COVID-19+HRV	2	2.11
	HADV+HRV	1	1.05
	HADV+MP	2	2.11
	229E+HRV	1	1.05
	PIV2+RSV	1	1.05
	RSV+MP	13	13.68
Triple viruses	HADV+HRV+MP	1	1.05
	HADV+RSV+MP	1	1.05
	PIV2+RSV+MP	2	2.11

Supplementary Table S3：Positive samples with respiratory pathogens confirmed by both the MPA assay and NGS.
